# In Situ Cancer Vaccination and Immunovirotherapy Using Oncolytic HSV

**DOI:** 10.3390/v13091740

**Published:** 2021-08-31

**Authors:** Nusrat Jahan, Shanawaz M. Ghouse, Robert L. Martuza, Samuel D. Rabkin

**Affiliations:** 1 Molecular Neurosurgery Laboratory and Brain Tumor Research Center, Department of Neurosurgery, Massachusetts General Hospital, Harvard Medical School, Boston, MA 02114, USA; NJAHAN@mgh.harvard.edu (N.J.); shanawazghouse81@gmail.com (S.M.G.); RMARTUZA@mgh.harvard.edu (R.L.M.); 2 Department of Neurosurgery, Massachusetts General Hospital, 185 Cambridge St., CPZN-3800, Boston, MA 02114, USA

**Keywords:** oncolytic virus, herpes simplex virus, immunotherapy, cancer vaccine

## Abstract

Herpes simplex virus (HSV) can be genetically altered to acquire oncolytic properties so that oncolytic HSV (oHSV) preferentially replicates in and kills cancer cells, while sparing normal cells, and inducing anti-tumor immune responses. Over the last three decades, a better understanding of HSV genes and functions, and improved genetic-engineering techniques led to the development of oHSV as a novel immunovirotherapy. The concept of in situ cancer vaccination (ISCV) was first introduced when oHSV was found to induce a specific systemic anti-tumor immune response with an abscopal effect on non-injected tumors, in the process of directly killing tumor cells. Thus, the use of oHSV for tumor vaccination in situ is antigen-agnostic. The research and development of oHSVs have moved rapidly, with the field of oncolytic viruses invigorated by the FDA/EMA approval of oHSV talimogene laherparepvec in 2015 for the treatment of advanced melanoma. Immunovirotherapy can be enhanced by arming oHSV with immunomodulatory transgenes and/or using them in combination with other chemotherapeutic and immunotherapeutic agents. This review offers an overview of the development of oHSV as an agent for ISCV against solid tumors, describing the multitude of different oHSVs and their efficacy in immunocompetent mouse models and in clinical trials.

## 1. Introduction

**Figure 1:**
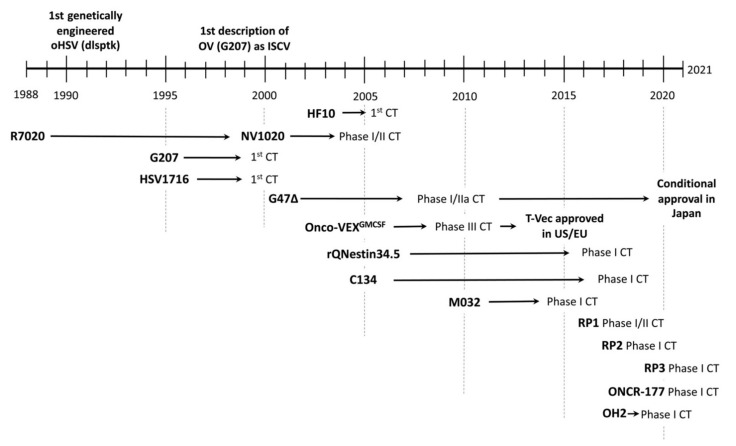
Development of oHSV: from discovery to clinical trial (CT) and approval as oncolytic agent. The center of the text corresponds to the year.

Cancer is a multi-step process that requires genetic and epigenetic changes to transform a normal cell into a cancer cell. However, tumors are complex organs consisting not only of cancer cells but a tumor microenvironment (TME) containing recruited ‘normal’ cells such as innate and adaptive immune cells, vasculature and lymphatics, and stromal cells [1]. The TME is often tumor-promoting and immunosuppressive, playing a large role in therapeutic resistance. The traditional modalities used to treat cancer (surgery, radiotherapy, and chemotherapy) are associated with adverse effects and often fail to eradicate cancer. Current immunotherapies targeting the TME are usually only effective in tumors with a high mutational load and/or tumors that are so-called immunologically “hot”, but are less effective in “cold” tumors [2]. Therefore, new treatment modalities for cancer are needed. Oncolytic viruses (OVs) are a distinct class of anti-cancer agents, first evaluated in the 1950s [3], with unique mechanisms of action: (i) selective replication in and killing of cancer cells while sparing normal cells and tissue, and amplifying in situ and spreading through the tumor; and (ii) exposing tumor antigens and promoting anti-tumor immune responses (immunovirotherapy). An oncolytic herpes simplex virus (oHSV) was the first genetically-engineered OV [4], and this class of OVs has remained at the forefront of the field [5]. When oHSVs were initially tested in syngeneic tumors in immunocompetent mice, it was found that they had properties of in situ cancer vaccination (ISCV), where induction of immunogenic cancer cell death and tumor inflammation in situ leads to priming and activation of anti-tumor immunity [6,7]. To date, fourteen oHSVs have been translated to the clinic [8] (Figure 1). The oHSV talimogene laherparepvec (T-VEC, Imlygic^TM^) was the first OV approved by the FDA in the USA in 2015 and the EMA in Europe [5]. Its approval has validated the OV modality and increased the effort to develop more effective oHSVs and combination strategies. In this review we discuss the development of oHSV as a novel immmunovirotherapy agent for ISCV against solid tumors, and strategies to enhance its efficacy. We have focused our discussions on oHSV studies evaluating immune responses in immunocompetent tumor models.

## 2. Oncolytic Viruses (OVs) and In Situ Cancer Vaccination (ISCV)

OVs are replication-competent viruses of either natural origin or genetically-engineered to selectively infect, replicate in, and kill cancer cells, but not normal cells [9]. Many pathogenic and non-pathogenic DNA and RNA viruses have been developed as OVs, and shown significant therapeutic promise preclinically and for some patients in clinical trials [9,10]. Immunovirotherapy, OV-mediated immunotherapy of cancer, is at the forefront of these investigations due to advances in our understanding of tumor and virus immunology [9,10,11]. Arming OVs by inserting therapeutic transgenes into the viral genome for localized expression in the tumor combines gene therapy with virotherapy and can alter an immune-suppressive TME into an immunologically active one [12]. Delivery routes for OVs depend on location, accessibility, the number of tumor sites, virus properties, elimination by the patient’s immune system, and potential toxicity; hence, multiple routes have been investigated for oncolytic virotherapy [13]. Among these, intratumoral delivery is the most commonly employed, as OVs can be targeted directly to the tumor site without dilution, neutralization, or deleterious effects to other organs.

**Figure 2:**
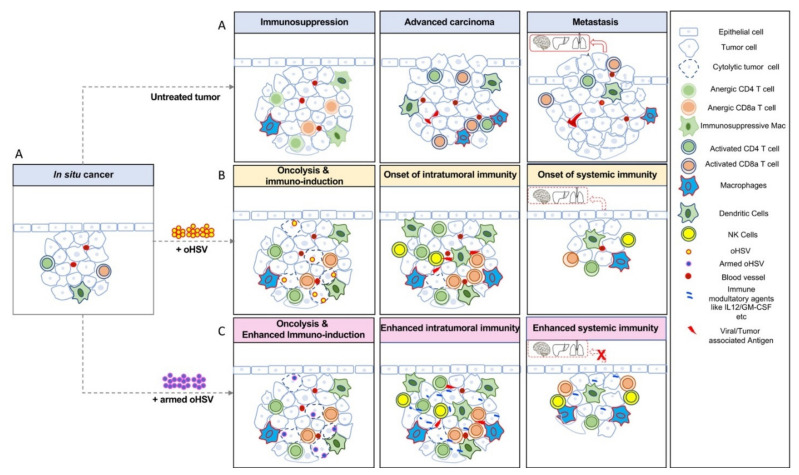
Oncolytic herpes simplex viruses (oHSVs) refine anti-tumor immune responses in cancer. (**A**) In situ carcinoma, localized cancer confined to the epithelial layer, where normal cells transition into cancerous cells. In untreated conditions, in situ cancer progresses to invasive cancer, where cancer cells acquire specific capabilities, transform to become invasive, and escape into the blood stream to establish new tumors in distant organs such as the lungs, brain, and liver, etc., through metastasis. (**B**) In situ cancer vaccination (ISCV) is induced by oHSV infection. oHSV induces cytolysis and cell death, as well as the release of viral antigens and tumor-associated antigens (TAAs), leading to the recruitment of innate immune cells such as NK cells and antigen-presenting cells (macrophages and DCs). Cytokines and chemokines released during this immune response enhance systemic immunity, recruiting effector T cells as critical components of the tumor microenvironment (TME). Primed T cells efficiently remove tumor cells, thereby preventing metastasis. (**C**) Tumors treated with armed oHSVs expressing immune modulatory genes such as IL12, GM-CSF, etc., often generate more efficient anti-tumor immune responses, even in highly immunosuppressed tumors such as glioblastoma and prevent metastasis, for example, in triple negative breast cancer.

Once the OV or oHSV arrives at the tumor, infection results in immunogenic cell death, as well as the release or exposure of viral antigens or pathogen-associated molecular patterns (PAMPs) and tumor-associated antigens (TAAs), including neoantigens and danger-associated molecular patterns (DAMPs), such as ATP, HMGB1, calreticulin, and an inflammatory response [14,15,16]. This in turn sends pro-inflammatory signals, which recruit NK cells and professional antigen-presenting cells (APCs), mostly dendritic cells (DCs) and macrophages to the infection site, ultimately priming and activating tumor-specific T cells [17,18,19]. The activated T cells proliferate and travel to the TME and exert their effector functions against cancer cells (Figure 2). During this process, cytokines and chemokines are released by the activated immune cells and collectively these events create an immune-inflamed site with strong inflammatory responses [20], and induce ISCV [21,22]. The first description of OV-induced ISCV was with oHSV G207 in 1998 [6,23]. These studies revealed that a priori knowledge of the identity of the tumor antigens unique to a given patient/tumor is not required for the induction of a systemic anti-tumor immune response and is therefore considered “antigen-agnostic” immunotherapy [21]. ISCV acts locally at the virus-infected tumor and at distal lesions (abscopal effect) [22]. ISCV is often studied using (i) bilateral tumor models and the evaluation of abscopal effects on non-injected tumors, as oHSV does not typically spread systemically or between distal tumors [24], and (ii) tumor rechallenge experiments after OV treatment has caused tumor elimination or ‘cure’. The immunocompetent mouse cancer cell line tumor models described below are indicated in Table 1.

## 3. Herpes Simplex Virus (HSV) as a Candidate for the Treatment of Cancer

**Figure 3:**
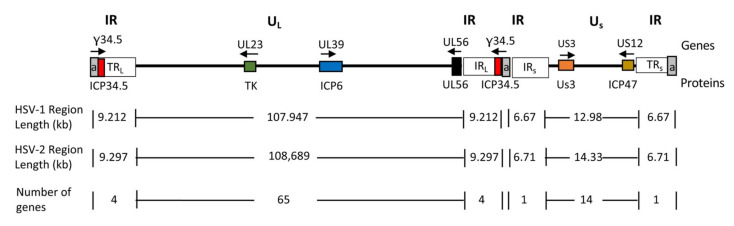
Schematic diagram of HSV genome. The genome consists of unique long (U_L_) and short (U_S_) sequences bracketed by terminal (TR_L_ and TR_S_) and internal (IR_L_ and IR_S_) inverted repeat (IR) sequences. The number of *a* sequence repeats (a, in gray) is variable. Genes relevant for tumor-specificity are shown as colored boxes and their names are indicated above the genome line; the gene product names are indicated below the genome line. Arrows indicate the direction of transcription.

HSV-1 and HSV-2 are closely related human neurotropic herpesviruses from the family Herpesviridae. The virion is composed of a capsid core, tegument, and envelope containing viral glycoproteins. The genomic DNA lengths of HSV-1 and HSV-2 are about 152 and 155 kb, respectively, and are structurally composed of two unique regions, a unique long (U_L_) and a unique short (U_S_) flanked by inverted repeats (R_L_ and R_s_) (Figure 3) [64]. The genome encodes approximately 84 gene products, with most present as single-copy contiguous transcription units, except for those present within the inverted repeats and diploid (α0, α4, γ34.5) or spliced (α0, UL15, α22, α47, γ34.5) [64]. During the past three decades, HSV has advanced from a human pathogen to a promising cancer therapeutic (oHSV) due to the improved understanding of its genetics and biology, and our capacity for genetic engineering [65]. This has allowed the construction of oHSVs seamlessly modified with increased tumor-targeting specificity, decreased off-target toxicity, and anti-tumor activity. Although the majority of oHSV constructs are in HSV-1, some are in HSV-2 [65]. Several properties of HSV make it an attractive choice as an oncolytic agent against various cancers: (i) the function of most gene products has been characterized and many viral genes are non-essential for virus replication and thus can be deleted to accommodate large or multiple transgenes (~30 kb) without dramatically affecting virus production in cultured cells; (ii) the virus DNA does not integrate into host chromosomes so the risk of insertional mutagenesis is minimal; (iii) it is a lytic virus that infects many different cell types and species with high efficiency; (iv) the expression of transgenes is tolerated; and (v) the availability of anti-HSV drugs (e.g., valacyclovir, acyclovir, ganciclovir) provides additional safety against any undesired infection or replication in treated patients. It is important to note that HSV replicates more poorly in mouse compared to human cells, so the activity observed in immunocompetent mouse tumor models does not represent the level of virus replication and cytotoxicity likely to be seen in patients.

## 4. Generation of oHSV

**Figure 4:**
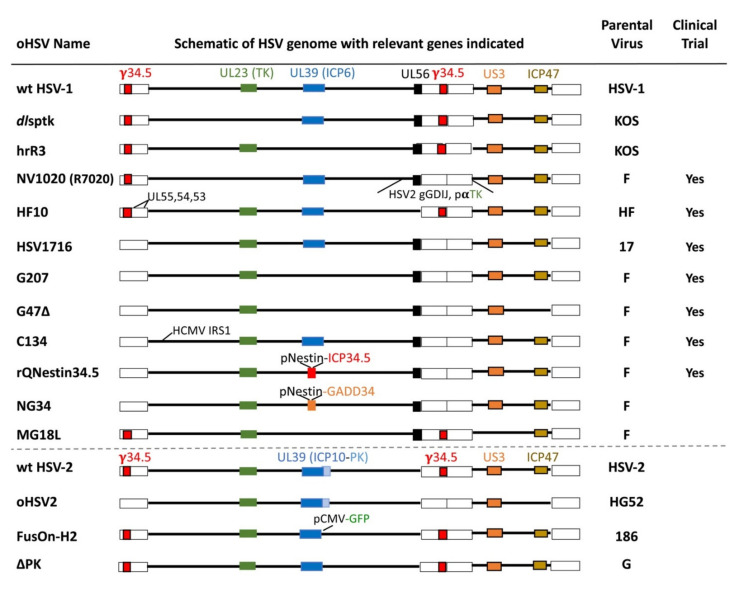
The major un-armed oHSVs with important gene modifications indicated. Abbreviations: wt, wild type; PK, protein kinase; gGDIJ, glycoproteins G, D, I, J; pαTK, α promoter driving TK; pNestin, nestin promoter-enhancer; pCMV, CMV promoter; and GFP, green fluorescent protein.

The key considerations in oHSV design are to ensure its: (i) safety, so the virus only grows in targeted tumor cells, sparing normal cells, and is non-pathogenic, and (ii) oncolytic activity, for selective virus replication and cell death to deliver maximum anti-tumor efficacy. The generation of oHSV has been achieved by means of genetic engineering, except for spontaneously arising HF10 [65,66]. Major oHSVs without therapeutic transgenes are listed in Figure 4. The timeline of the path of oHSVs towards clinical trials over the last three decades is presented in Figure 1.

The generation of oHSV using recombinant DNA technology includes (i) introducing changes (deletions/mutations) in viral genes that are non-essential in cancer cells for oHSV cancer specificity, such as genes necessary for replication in non-dividing cells, blocking innate antiviral responses, or blocking apoptosis, which can also decrease replication [65], and (ii) modifications of viral glycoproteins to retarget recombinant virus entry via cancer-associated surface molecules instead of the endogenous receptors (tropism retargeting) [11]. The classical approach using recombinant DNA technology is based on homologous recombination in eukaryotic cells, where co-transfection of HSV viral genomes and a plasmid carrying the desired transgene flanked by virus sequences generates recombinant oHSV vectors [67]. Although this conventional approach is tedious, with a low recombination frequency, it can be improved by incorporating a reporter gene for the detection of recombinants. More recently, CRISPR/Cas9 technology has been used to create double-strand breaks and insertions or base modifications at targeted sites in the viral genome [68,69]. An alternate approach is using HSV bacterial artificial chromosomes (BACs), where the desired transgenes are introduced into the virus genome by homologous recombination in bacteria [70,71]. BAC systems result in the rapid generation of recombinant oHSV vectors, but BAC sequences should be removed, and mutations can occur during growth in *E. coli*, reducing virus replication.

### 4.1. Spontaneously Arising oHSV-1

HF10 (Canerpaturev or C-REV, TBI-1401) is an attenuated, replication-competent, spontaneously mutated oHSV with large deletions and insertions, which leads to a lack of expression of UL43, UL49.5, UL55, UL56, and latency-associated transcript (LAT) [66,72]. The loss of UL56 attenuates neuroinvasiveness and LAT reduces reactivation from latency [72]. HF10 has been examined in multiple tumor models to test its oncolytic and immune efficiency [72]. In a mouse syngeneic colon cancer model (MC26), HF10 injection of a subcutaneous tumor inhibited liver and peritoneal metastases and induced MC26-specific CTL in vitro [30]. HF10 inhibited the growth of both injected and non-injected tumors in a bilateral mouse M3 melanoma model, and protected mice cured of intraperitoneal tumors from tumor rechallenge [37]. In a mouse squamous cell carcinoma model (SCC-VII), HF10 significantly reduced tumor growth despite increased PD-L1 expression on tumor and immune cells, and increased CD8^+^ PD-1^−^ TILs in both injected and non-injected tumors [52]. HF10 inhibited tumor growth and induced CD8^+^ T cell infiltration in head and neck and oral squamous cell carcinoma [73,74]. These studies illustrate the robust immune-stimulating properties of oHSV.

Seven phase I and II clinical trials of HF10 have been conducted or are active to test its safety and efficacy against advanced solid tumors [75]. HF10 induced a cytotoxic immune response against recurrent breast cancer [76], and in a pancreatic ductal carcinoma trial HSV^+^ tumor cells were present at autopsy, as well as significant increases in CD8^+^ T cells and macrophages compared to control specimens [77].

### 4.2. oHSV-1 with Single Genetic Modifications

A series of investigations during the pre-oHSV era resulted in some important HSV-1s with single gene mutations [78,79,80,81,82]. These mutants were repurposed as first-generation oHSVs, due to their restricted replication in non-dividing cells and lack of pathogenicity. They included mutations in the following genes: (i) UL23, encoding thymidine kinase (TK) [80]; (ii) UL39, encoding ICP6 (the large subunit of ribonucleotide reductase) [78]; (iii) γ34.5 (RL1), encoding ICP34.5 (neurovirulence factor, blocks translation inhibition) [81]; and (iv) Us3, encoding a serine-threonine protein kinase (anti-apoptotic) [82,83] (Figure 4).

TK synthesizes nucleotide precursors for HSV DNA replication and thus is essential for virus growth in non-dividing/post-mitotic cells both in vitro and in vivo [84], prompting its evaluation as an oHSV. The first genetically-engineered OV was oHSV *dl*sptk (Figure 4), containing a deletion in the TK gene, which selectively killed glioma cells and increased the survival of nude mice bearing human orthotopic gliomas [4]. This ignited a search for OVs derived from other genetically-engineered viruses such as adenovirus E1B-deleted *dl*1520 (Onyx-015) [85] and opened the door to new generations of oHSVs, using other non-essential genes [65]. However, TK is required for sensitivity to anti-HSV nucleoside analog drugs, (i.e., valacyclovir, acyclovir, ganciclovir) [86], nullifying an important safety feature for any oHSV-associated toxicity in treated patients, which drove the search for additional non-essential HSV genes. ICP6 is also involved in nucleotide metabolism and required for replication in non-cycling cells and pathogenicity [65]. In contrast to TK, UL39 mutants are hypersensitive to nucleoside analogs (i.e., acyclovir) [87]. HrR3, an HSV-1 mutant with a LacZ gene insertion disrupting ICP6 [78] (Figure 4), showed oncolytic activity in human xenografts [87]. In a mouse colon cancer intraperitoneal model (MC26), intraperitoneal administration was much more effective than intravenous administration at inhibiting tumor growth [31].

ICP34.5 has multiple properties that make its deletion a common feature of oHSVs, especially for use in the brain; it overcomes the shutdown of protein synthesis through dephosphorylation of eIF2α, blocks autophagy by binding beclin 1, blocks IRF3 activation and IFN signaling by disabling STING and RIG-I, and is required for virus neuropathogenicity [65,88]. A γ34.5 mutant deleted for the amino-terminal 146 aa (ΔN146) replicated selectively in cancer cells, induced IRF3, and inhibited mouse breast tumor (4T1) growth and metastases [46]. Both R3616 and HSV1716 (Seprehvir) have deletions in both copies of γ34.5 in different HSV-1 strains [81,89]. R3616 inhibited tumor growth in glioma cell implants in immunodeficient mice [90,91]. HSV1716 inhibited mouse intracranial Harding–Passey melanoma, with HSV staining 7 days post-injection restricted to the tumors [38]. In mouse orthotopic breast cancer models HSV1716 inhibited tumor growth and metastases after intravenous administration, which was associated with increased activated CD8^+^ T cells, decreased Tregs, and the reprogramming of tumor-associated macrophages (TAMs) to a pro-inflammatory M1-like phenotype [45]. It was the first oHSV to enter clinical trials in Europe in a phase I trial for patients with recurrent glioma [92]. Out of eight glioblastoma (GBM) patients treated intratumorally, seven were seropositive and four survived over 14 months, with no adverse events [92]. In a third glioma trial, patients (six recurrent and six newly diagnosed) had virus injected into the brain adjacent to their resection cavity [93]. HSV1716 has also been in clinical trials for melanoma [94], mesothelioma [95], oral squamous cell carcinoma [96], and more recently in children and young adults with non-CNS solid tumors administered intratumorally [97] or intravenously [98], with six of eight and five of five seronegative patients seroconverting. Seroconversion indicates a functional adaptive immune response.

Us3 kinase activity overlaps the substrate specificities of PKA and Akt, phosphorylating p65, IRF3, IFNγRα, etc., to affect many antiviral pathways, as well as inhibiting apoptosis, which is frequently dysfunctional in tumor cells [65]. Us3 deletion mutant R7041 is tumor-cell-selective, with enhanced apoptosis in normal cells and inhibited tumor growth after intratumoral or intravenous delivery [99]. Although it was safe after systemic delivery in the periphery, it was not safe in the brain after intracerebral inoculation [100]. A Us3 deleted HSV-2 mutant has also been constructed [101].

### 4.3. oHSV-1 with Multiple Genetic Modifications

The demonstration that single-gene mutated HSV worked as a therapeutic agent against cancer prompted the development of multi-gene mutated recombinants with enhanced safety and efficacy. The first multi-mutated, second-generation oHSV was G207, with deletions at both γ*34.5* loci and a *lacZ* gene insertion in the *ICP6* gene (Figure 4) [102]. Safety is a particular concern when treating brain tumors, as the brain is a major potential site of clinical morbidity and mortality [103]. G207 was extremely safe after intracerebral injection in HSV-sensitive mice and nonhuman primates [104,105]. G207 is efficacious against most human cancer cell lines and solid tumors in preclinical animal models [106]. Studies with G207 provided the first demonstration of ISCV by an OV [23], illustrating some of the experimental approaches supporting ISCV. In immunocompetent mice, G207 inhibited the growth of treated mouse subcutaneous tumors (CT26 and M3 (Table 1)), as well as contralateral non-injected tumors, but not in immunodeficient mice, through the induction of tumor-specific CD8^+^ CTLs [6]. In mice with both subcutaneous and intracerebral tumors, injection of subcutaneous tumors (N18) also inhibited the growth of non-injected intracerebral tumors, with no evidence of virus spread, and provided protection against tumor rechallenge in the brain or periphery [24]. In three syngeneic liver metastasis models with mouse CT26 colon cancer cells, intratumoral injection of G207 significantly reduced the number of mice without liver metastasis, which did not occur in nude mice [25].

As most individuals have been exposed to HSV-1 and are seropositive, it was important to determine the effect of prior immunization of mice with HSV-1 on anti-tumor efficacy; intratumoral injection of G207 in N18 and CT26 tumors [32], intraperitoneal injection of HSV1716 for intraperitoneal tumors [107], and intrahepatic artery delivery of G207 or NV1020 (Figure 4) to liver metastases (CT26) were not affected, whereas intravenous delivery of low-dose NV1020 was modestly reduced [26]. In one study, pre-immunization increased survival times of mice with intracerebral melanoma after intratumoral HSV1716 treatment [36].

G207 was the first oHSV to enter clinical trials in the USA [108] and was found to be safe with potential efficacy when used alone or in combination with radiotherapy [108,109,110]. Recently, a phase I clinical trial of G207 in children with high-grade glioma reported encouraging results, with clinical responses seen in 11 of 12 patients [111]. Interestingly, three patients who seroconverted after high dose G207, a sign of immune response, experienced an overall survival of 18 months. Importantly, in four patients with post-treatment biopsies, post-treatment tissues showed an infiltration of TILs (CD4^+^ and CD8^+^) that increased over time compared to matched pre-treatment tissue, converting ‘cold’ tumors to ‘hot’ tumors, with no evidence of HSV-1 staining [111]. A second G207 clinical trial in children with recurrent or refractory cerebellar brain tumors is ongoing (NCT03911388) [112]. MGH1, comparable to G207, has been cloned into a BAC plasmid to enable the rapid construction of recombinant oHSVs, and the rescued oHSV is termed rHsvQ1 [113].

The Us3 mutant R7040 contained γ34.5 and was not safe enough in the brain, so an inactivating LacZ insertion in ICP6 was inserted to make MG18L (Figure 4), which was now safe in the brain and replicated selectively in cancer cells [100]. MG18L also synergized with PI3K/Akt inhibitors in killing human GBM stem-like cells in vitro and inhibiting tumor growth, which was associated with increased apoptosis [100]. Its activity in immunocompetent mice remains to be determined.

γ34.5Δ oHSVs (R3616, HSV1716, G207) replicate very poorly in patient-derived GBM stem-like cells, due to a block in true late gene translation, but did replicate in patient-matched serum-cultured GBM cells in vitro [114,115,116]. Because of the attenuated growth of γ34.5-deleted oHSV, even in cancer cell lines, there was an effort to enhance its activity safely through a number of genetic modifications. A screen for second site suppressor mutations of γ34.5Δ identified a deletion of Us12, encoding ICP47, that restored virus replication in non-permissive cancer cells without affecting attenuated neurovirulence [117,118]. Deletion of ICP47 places the late Us11 gene (PKR inhibitor) under the control of the immediate-early ICP47 promoter, which blocked protein shut-off [119]. Introduction of this deletion into G207 created the third generation oHSV, G47Δ (Teserpaturev; Delytact^®^) (Figure 4), which replicated better in vitro, including in GBM stem-like cells [114,115,116], and more effectively inhibited tumor growth [33]. As MHC I antigen presentation is blocked by ICP47 in HSV-infected cells (not in rodent cells), viral and tumor antigens are more efficiently presented on cancer cells infected with G47**Δ**, resulting in the enhanced activation of human lymphocytes compared with G207 [33]. In preclinical studies using several cancer types and experimental models, G47Δ exhibited robust antitumor efficacy in human cancer stem-like cells, including GBM [114,120,121,122].

G47Δ was more efficacious than G207 in inhibiting the growth of neuroblastoma and breast cancer syngeneic subcutaneous tumors [33,41]. In a transgenic breast cancer model (C3(1)/T-Ag), G47Δ injection of the first spontaneously-arising mammary tumor significantly inhibited tumor progression and intratumoral treatment of mouse syngeneic breast cancer cell (M6c) brain implants significantly extended survival [41]. In a HPV^+^ cervical cancer model, G47Δ (T-01) inhibition of tumor growth was associated with an increase in CD8^+^ T cells in the tumor [54]. G47Δ treatment of a syngeneic bilateral hepatoma model inhibited both injected and non-injected tumors, increased tumor-specific IFNγ splenocytes in vitro, and significantly increased CD8^+^ but not CD4^+^ T cells [55]. G47Δ has undergone a number of early-phase clinical trials in Japan for recurrent glioma, castration-resistant prostate cancer, recurrent olfactory neuroblastoma, and malignant mesothelioma [123]. A single-arm phase II clinical trial for recurrent glioma was recently completed and met its 1-year survival rate endpoint, with 92% in the G47Δ cohort versus 15% in comparative historical controls. This led to its conditional approval in June 2021 by the Japanese Ministry of Health, Labor, and Welfare [124]. A similar combination of γ34.5 and ICP47 deletions is present in OncoVex, the parental virus of T-Vec [59].

Other notable genetic modifications to enhance γ34.5-deleted oHSV include driving γ34.5 with a cancer cell specific promoter or expressing a complementing protein. rQNestin34.5 was constructed with γ34.5 expression driven by the glioma-selective enhancer Nestin in rHsvQ1, a G207 analog [125]. It was much more efficacious in vitro and inhibiting tumor growth in human glioma brain xenografts than rHsvQ1 [125]. A phase I clinical trial for GBM with rQNestin34.5 is currently ongoing (NCT03152318) [126]. A similar strategy was used in KeM34.5, where γ34.5 is driven by a different glioma-selective promoter, Musashi1 in G207 [127]. KeM34.5 was safe after intracerebral injection in mice, replicated in glioma cell lines about 2 logs better than G207, and extended survival of mice with human gliomas about 2-fold [127]. An alternate strategy is to express a mammalian or viral ortholog in place of γ34.5. The C-terminal domain of γ34.5, involved in blocking host protein synthesis shutoff, has structural and functional homology to human GADD34 and mouse MyD116 [128]. NG34 is identical to rQNestin34.5 (Figure 4), except GADD34 is expressed in place of γ34.5 [129]. It had similar in vitro and in vivo efficacy as rQNestin34.5 but was less neuropathogenic [129]. In GD116, a fusion between the N-terminus of γ34.5 and C-terminus of MyD116, was inserted into ICP6 in G47Δ, which replicated better in human breast cancer cells than GD-empty [130]. Human cytomegalovirus (HCMV) IRS1, a PKR evasion protein functionally analogous to γ34.5 was inserted into γ34.5-deleted R3616 to construct C134 [131]. In mouse Neuro 2A and DBT intracerebral models, C134 was more efficacious than R3616, whereas it lacked efficacy in immune-deficient mice [34]. C134 was safe after intracerebral injections in mice and non-human primates [132], and is currently in phase I clinical trial in patients with recurrent GBM (NCT03657576).

### 4.4. Intertypic HSV Recombinant

NV1020 (R7020) is an HSV-1/HSV-2 intertypic recombinant, with a deletion in the IR_L_ region resulting in loss of U_L_56 and one copy of γ34.5, which is replaced with a fragment from HSV-2 encoding gG, gJ, gD, and gI glycoproteins (US2 to US8) (Figure 4), that was initially constructed and tested as a vaccine against HSV-1 and HSV-2 [79]. NV1020 was examined and found to be efficacious in a number of different tumor types—colorectal, pancreatic, mesothelioma, rhabdomyosarcoma, and liver metastases [133]. This led to two clinical trials for patients with refractory hepatic metastases from colorectal cancer [134,135,136,137]. The first clinical trial, dose-escalating NV1020 administered in a single hepatic artery infusion followed 28 days later by standard chemotherapy, was the first study of intravascular oHSV in patients [135]. After virus treatment, levels of carcinoembryonic antigen (CEA) significantly decreased, with a further decrease after chemotherapy, and no dose-limiting toxicities [137]. In the second phase I/II trial, patients received four weekly infusions of NV1020, followed by chemotherapy, with fourteen of twenty-two patients at the optimal biological dose showing stable disease (SD) [134]. Interestingly, ~50% of patients exhibited ‘progressive disease’ (PD) at 1 month, which then regressed, so 64% of those were classified as SD at 6 months [136]. This pseudoprogression, indicative of an immune response, has been seen in many OV clinical trials [138] and with other immunotherapies [139]. NV1023 has a lacZ insertion into ICP47 of NV1020 and behaved similarly to NV1020 in inhibiting mouse SCC-VII tumor growth [53]. In a transgenic model of prostate cancer (TRAMP), systemic administration of NV1023 significantly inhibited primary prostate tumor growth and lymph node metastases [140]. When TRAMP-C2 cells were implanted bilaterally, NV1023 only inhibited the growth of the injected and not non-injected tumors [141].

### 4.5. oHSV-2 with Gene Modifications

The HSV-2 gene for the large subunit of ribonucleotide reductase (ICP10) has a protein kinase (PK) domain not present in HSV-1. ICP10PK activates Ras signaling and is required for growth in normal cells, and blocks multiple programmed cell death pathways [142]. ΔPK contains a deletion of the N-terminal PK domain of ICP10 (C-terminal ICP6 counterpart) in HSV-2 strain G (Figure 4). Infection of human melanoma and breast cancer cells induced programmed cell death (PCD), including apoptosis; calpain activation; pyroptosis; and autophagy; inhibited IL-10, IL-18, and CTLA-4 expression; and induced the secretion of inflammatory cytokines such as TNF-α and IL-6 [142]. FusOn-H2 has a similar deletion of ICP10PK, but replaced with GFP, in HSV-2 strain 186 [143]. It inhibited orthotopic bladder tumors, orthotopic breast tumors and lung metastasis, and Neuro-2A tumor growth both in injected and contralateral sites to a significantly greater extent than oHSV-1 Baco-1 (γ34.5Δ), while inducing more active tumor-specific CTL and IFNγ secretion [44,58,144]. In nude mice, FusOn-H2 inhibited Neuro-2A tumor growth to a similar extent as in immunocompetent mice; however, there was no effect on contralateral tumors, whereas the adoptive transfer of splenocytes from FusOn-H2 treated mice inhibited tumor growth [144].

oHSV2 was constructed in wild-type HSV-2 strain HG52 with both γ34.5 and ICP47 genes deleted and inhibited 4T1 tumor growth to the same extent as oHSV1 (γ34.5Δ, ICP47Δ) [42] (Figure 4). This anti-tumor activity was associated with an elevation of NK cells and a mild decrease of Tregs in the spleen [42]. oHSV2 was efficacious against a treated subcutaneous CT26 tumor and demonstrated a systemic immune response against non-injected tumors, which was associated with increased NK, CD8^+^ T, and dendritic cells in the tumor [19]. Us3 of HSV-2 and HSV-1 have many similar functions and one of them is prevention of apoptosis. Us3-deleted HSV-2 (L1BR1) combined with chemotherapy increased apoptosis in L1BR1, but not wild-type, infected cancer cells and alone showed oncolytic activity in implanted human SW1990 pancreatic tumors [101]. When compared to HSV-1 Us3 deletions, HSV-2 Us3 deletions are only minimally attenuated for neurovirulence [145].

### 4.6. Receptor-Retargeted oHSVs

Another strategy to engineer oHSVs involves retargeting them to tumor-specific receptors and de-targeting them from virus receptors through the insertion of a foreign ligand into viral gD, gH or gB, so that the virus exhibits target-specific inhibition of human tumor growth [146]. The first fully retargeted oHSV used a receptor ligand for targeting; R5141 contained a chimeric IL-13-gD protein to bind overexpressed IL-13 receptor α2 (IL-13Rα2) on GBM [147]. An alternate strategy is to use anti-target single chain variable fragments (scFvs), for example, targeting HER2, EGFR, PSMA, or EpCAM fused to the N-terminus of gD, gB, or gH [148]. A novel strategy to screen antibodies to identify new targets/cell surface antigens for retargeted oHSV has been described [149]. It is important to note that many of these scFvs/antibodies recognize only the human receptor, complicating safety studies or requiring cancer cells transduced with the human target for studies in immunocompetent mice, such as with syngeneic BALB/c-HGG-HER2 glioma cells [150].

## 5. Armed oHSV

A striking advantage of using oHSV for ISCV is its capacity to incorporate large or multiple transgenes (up to ~30 kb) within the viral genome. Expressing transgenes from oHSV-infected cancer cells enables local targeting of other cells in the TME, which multiplies the capabilities of oHSV to also act as a gene therapy vector [12]. Arming oHSV with various immune-modulatory genes has been shown to increase the efficacy of oHSV in various cancer models and is a component in the only oHSV (T-VEC) currently approved by the FDA at the time of this writing [5]. For potent cytokines or other gene products, localized expression can significantly reduce toxicity arising from systemic administration, as well as increasing their concentration within the tumor.

### 5.1. oHSVs Armed with Granulocyte-Macrophage Colony Stimulating Factor (GM-CSF)

GM-CSF is a growth factor first identified as an inducer of differentiation and proliferation of granulocytes and macrophages from hematopoietic progenitor cells that is mainly produced by T cells, B cells, epithelial cells, and fibroblasts upon activation, and acts in recruiting and activating antigen presenting cells (DCs, macrophages, MDSCs) [151]. In the mouse B16 melanoma model, irradiated tumor cells expressing GM-CSF, acted as a vaccine, inducing potent, long lasting T cell dependent tumor-specific immunity [152], identifying it as a promising immune modulatory candidate for arming oHSVs. GM-CSF was thus one of the first cytokines to be cloned into oHSV, including in NV1034 and OncoVEX^GMCSF^ [53,59]. The effects of GM-CSF expression from oHSV in syngeneic mouse tumor models were modest [59,141]. OncoVEX^GMCSF^, renamed talimogene laherparepvec (T-VEC; Imlygic^TM^), with γ34.5 replaced with the human GM-CSF gene under the control of the CMV promoter, was the first armed oHSV to enter clinical trials, treating patients with cutaneous or subcutaneous solid tumors [153].

Successful clinical trials made T-VEC the first OV to be FDA- and EMA-approved [5]. The pivotal phase III OPTiM clinical trial in patients with advanced melanoma (unresectable stage IIIB-IV) compared intratumoral T-VEC with subcutaneous GM-CSF and resulted in a complete response rate (CR) of 16.9% versus 0.7% and a median survival of 24.5 versus 18.9 months [154]. T-VEC’s mechanism of action was revealed at the single-cell level in a study in primary B cell lymphoma, where T-VEC transcripts were observed 24 hrs after injection in malignant and non-malignant cells in the injected, but not the non-injected lesions [155]. T-VEC activated the interferon pathway and induced a rapid influx of innate immune cells, followed by increased cytotoxic T cells and decreased Treg cells [155]. In a recent phase II clinical study, where the CR was 14%, there was a 2.4-fold median increase in CD8^+^ T-cell density in non-injected lesions 6 weeks after two T-VEC doses, and a significant increase in CD8^+^ effector and memory TILs, indicative of systemic immune effects [156]. T-VEC is currently in clinical trials for pancreatic cancer, non-melanoma skin cancer, breast cancer, and sarcoma. OH2, oHSV-2 expressing GM-CSF with a similar structure to that of T-VEC, has entered clinical trials in China for metastatic solid tumors with injectable lesions (NCT04386967) [157].

### 5.2. oHSVs Armed with Interleukin 12 (IL-12)

IL-12 is a heterodimeric master regulator of the immune system, primarily responsible for cell-mediated immunity, with diverse functional effects, including growth/activity of NK, T, and B cells; differentiation of Th1 cells; reprogramming immune suppressive cells (MDSCs, TAMs); stimulating the production of IFNγ; enhancing MHC I antigen presentation in tumor cells; and anti-angiogenesis [151,158]. Due to these diverse functional effects, significant antitumor activity was observed in many preclinical models [158]. Unfortunately, systemic administration of IL-12 or IL-12 transduced TILs in patients with advanced cancer was found to be exceedingly toxic [151]. Therefore, the localized delivery of IL-12, such as with armed oHSVs, is a promising strategy. A number of oHSVs expressing IL-12 have been generated (G47Δ-mIL12, M002, NV1042, R-115) that exhibited superior efficacy compared to their parental oHSVs not expressing IL-12 [159].

G47Δ-mIL12 expresses mouse IL-12 under the control of the ICP6 promoter. In an orthotopic GBM mouse model (005), intratumoral injections of G47Δ-mIL12 resulted in a significant survival extension compared to G47Δ-empty injections, with a significant reduction in Tregs and vascularity [47]. In the syngeneic 4T1 triple-negative breast cancer (TNBC) mouse model, G47Δ-mIL12 treatment significantly reduced the primary tumor burden and metastasis in both early and late stages of tumor development, in a CD8^+^-dependent fashion [43]. G47Δ-mIL12 treatment induced both local and systemic effects, including increased CD8^+^ TILs and reduced MDSCs in both treated and untreated tumors, increased DCs in the spleen, and decreased tumor vascularity and increased CXCL10 [43].

M002, containing an insertion of murine IL-12 under the control of the EGR-1 promoter in place of γ34.5, extended survival in an intracerebral Neuro-2a murine neuroblastoma model [35] and in a mouse glioma model (4C8) compared to parental oHSV R3659 [50]. In a spontaneous metastatic ovarian cancer mouse model (MISIIR-TAg mice), 81% of mice treated intraperitoneally with M002 were less likely to develop metastatic ovarian cancer when compared to 18% in PBS control groups, and this was associated with higher numbers of tumor antigen-specific CD8^+^ T-cells within the omentum and peritoneal cavity [160]. M032, a similar oHSV expressing human IL-12, was safe after intracerebral injection in HSV-sensitive non-human primates [161] and is currently in clinical trials for recurrent glioma (NCT02062827) [162]. Interestingly, M032 has also been evaluated in a canine clinical trial in pet dogs with glioma [163].

NV1042, derived from NV1020 and expressing murine IL-12, inhibited syngeneic squamous cell carcinoma (SCC) tumor growth compared to its parental NV1020 or NV1034 [53]. Depletion of CD4^+^ and CD8^+^ T cells abrogated the effect of IL-12 [53,164]. NV1042 delayed tumor progress in two spontaneously-arising transgenic tumor models—after intratumoral injection of breast cancer in C3(1)/SV40TAg mice and intravenous injection in TRAMP mice with prostate cancer [41,140].

R-115 is a HER2-retargeted oHSV, derived from R-LM113 and armed with murine IL-12. It was more efficacious in a syngeneic lung cancer model (LLC1-HER2) than R-LM113 and induced more robust immunity [165]. Treated mice that survived the primary tumor were protected from tumor rechallenge [165]. In a GBM mouse model, intratumoral R-115 treatment increased the number of CD4^+^ and CD8^+^ T cells infiltrating the tumor mass compared to R-LM113 [166].

### 5.3. oHSV Armed with IL-15

IL-15 stimulates CD8^+^ T cell proliferation and CTL, and induces proliferation and activation of NK cells [151]. An oHSV-2 expressing IL-15 was not significantly better at inhibiting CT26 tumor growth than oHSV-2-GFP [167]. However, OV-IL15C, expressing an IL-15 super agonist (human IL-15/IL-15Rα complex), from OV-Q1 (HSV-1 γ34.5 and ICP6 deleted; rHsvQ1) was more effective at extending survival in a CT2A syngeneic mouse GBM model than OV-Q1, and this was associated with increased in NK and T cell infiltration [27]. The combination with intratumoral injection of human EGFR-CAR NK cells further significantly extended the survival of mice with CT2A-hEGFR brain tumors [27], representing a promising therapeutic strategy.

### 5.4. oHSVs Armed with Immune Checkpoint Inhibitors (ICIs)

In light of the fact that oHSVs often induce PD-L1 expression in tumors, the reported combination effects with systemic immune checkpoint inhibitors (ICIs), and the toxicity of systemic ICIs, arming oHSVs with ICIs for local expression has therapeutic potential. NG34 (Figure 4) expressing scFvPD-1 (NG34scFvPD-1) cured ~40% of syngeneic mice with GL261N4 GBM tumors, but this was not significantly different from NG34, with no effect seen in nude mice [51]. In CT2A/PD-L1 GBM there was no significant increase in median survival. Although 17% of mice were cured, this was less than with anti-PD-1 antibodies [51]. Anti-PD-1 scFv was inserted into OVH (γ34.5 and ICP0 deleted) to generate OVH-aMPD1, and examined in a bilateral Hepa1–6 liver cancer model, where it was significantly better at inhibiting the tumor growth of both injected and non-injected tumors compared to OVH when large tumors were treated but not small ones. This was associated with a significant increase in activated T cells and a decrease in MDSCs [56]. The tumor growth inhibition of OVH-aMPD1 was the same as OVH + systemic anti-PD-1 [56]. oHSV2 (Figure 4) expressing anti-human anti-PD-1 mAb (oHSV2-aPD1) was examined in B16R cells (B16 expressing HSV receptor HVEM) in syngeneic mice expressing human PD-L1, where it significantly inhibited tumor growth, but not survival, compared to oHSV2 [40]. Treatment with oHSV2-aPD1 resulted in a significant increase in CD4^+^ and CD8^+^ T cells and a decrease in NK cells and macrophages in the spleen [40].

### 5.5. oHSVs Armed with Secreted Chimeric Molecules

Bispecific T cell engagers (BiTEs) are immunostimulatory bispecific antibodies, consisting of an anti-CD3ε scFv fused to a tumor-targeted antibody via a flexible linker that primarily activates T cells to kill tumor cells. In preclinical models, BiTEs have shown strong antitumor activity, superior to that of conventional monoclonal antibodies and other bispecific antibodies [168]. However, BiTEs have a limited capacity to penetrate tumor tissue, limiting their therapeutic potential in cancer immunotherapy. In addition, BiTEs are short lived and the continuous infusion of BiTEs can lead to increased toxicity. OHSVs expressing BiTEs can mediate tumor-targeted cytotoxicity locally within the TME. A BiTE targeting the pan-cancer antigen PD-L1 has been generated that crosslinks PD-L1-positive cells, and CD3ε on T cells was cloned into rHsvQ1. It triggers T cell activation and the release of proinflammatory cytokines such as IFNγ, IP-10, and TNFα [169]. In a human malignant ascites model, PD-L1 BiTE-expressing oHSVs activated intratumoral T cells, resulting in the depletion of tumor cells and M2-like macrophages [169]. Tumor cells in immunosuppressive ascites expressed higher levels of PD-L1, making them better targets for BiTEs. This approach activates endogenous T cells within malignant ascites, generates a proinflammatory milieu, and eliminates cells promoting tumor progression. Using oHSV for the local expression of PD-L1 BiTEs harnesses immunosuppressive protumor conditions to augment immunotherapy in immunologically ‘cold’ clinical cancers.

FusOn-PL is an armed version of FusOn-H2 (Figure 4), with a secreted chimeric molecule composed of a tumor-associated antigen (TAA) affibody and Protein L (PL), which binds immunoglobins and exposes Fc to Fc receptor binding [170]. In a murine CT26-HER2 colon tumor model, FusOn-PL induced NK cell infiltration, TAA-HER2-specific immune responses, and eliminated half of the tumors. Tumor-free mice were protected from rechallenge with CT26-HER2 tumors, indicating anti-tumor immunity [170].

### 5.6. oHSVs Armed with Multiple Transgenes

OHSVs can be armed with multiple transgenes to expand their antitumor activity. R-123, a modified version of R-115 (see 5.2) that expresses both IL-12 and GM-CSF, inhibited HER2-LLC tumor growth, with about 40% of responders, which was improved to 100% cures when combined with anti-PD-1 [171]. Depletion of CD4^+^ T cells or IFNγ abrogated combination efficacy, whereas anti-CD8 reduced responses to the level seen with R-123 alone, and NK cell depletion had only a modest effect [171]. VG161 encodes four transgenes (IL-12, IL-15, IL-15Rα, and PD-L1 blocker) in a backbone deleted for γ34.5 and the terminal repeat [60]. In a bilateral A20 model, mVG161 treatment only resulted in improved inhibition of non-injected tumors versus VG160 (no transgenes). Although tumor-infiltrating immune cells and tumor-specific IFNγ-expressing splenocytes in vitro were increased after mVG161 treatment in CT26 tumor-bearing mice, the differences compared to VG160 were not significant [60]. VG161 is currently in phase I clinical trials in China for patients with advanced solid tumors (NCT04758897) and liver cancer (NCT04806464).

RP1 is similar to T-VEC, with deletions of γ34.5 and ICP47 and expressing hGM-CSF, except in a different wt HSV-1 backbone, and in addition encoding fusogenic GALV-GP-R^-^ (not active in mice) [61]. In a rat bilateral 9L model, mouse (m)RP1 was significantly better than mRP1 without GALV at inhibiting non-injected tumors. Expression of anti-CTLA-4 (mRP2) further improved anti-tumor activity in the mouse A20 model, and combining mRP1 with anti-PD-1 antibody significantly improved the inhibition of non-injected tumors [61]. This led to the development of RP3, expressing two immune co-stimulatory ligands (4-1BBL and CD40L), in addition to anti-CTLA-4 and GALV-GP-R^-^. RP1 is currently in three clinical trials in cutaneous squamous cell carcinoma (NCT04349436) and combined with anti-PD-1 (NCT04050436), and advanced solid tumors and combined with anti-PD-1 (NCT03767348). RP2 is currently in clinical trials in advanced solid tumors and combined with anti-PD-1 (NCT04336241). A clinical trial evaluating RP3 alone and in combination with anti-PD-1 in patients with solid tumors has recently started (NCT04735978) (Figure 1).

ONCR-177 is armed with five human transgenes (hIL12, hFLT3LG (extracellular domain), hCCL4, and antagonists to hPD-1 and hCTLA-4) cloned into ONCR-159, which has normal cell-specific miRNA targets regulating the expression of HSV ICP4, ICP27, UL8, and γ34.5 [172]. mONCR-171 is identical to ONCR-177 except with mouse transgenes [29]. mONCR-171 significantly improved the response rate compared to ONCR-159 in bilateral A20 and B16-N1 syngeneic tumor models in both injected and non-injected tumors, whereas with MC38 it showed this effect only in injected tumors and with CT26 only in non-injected tumors [29]. Mice cured of A20 and CT26 tumors were protected from tumor rechallenge. Transcriptional analysis of mONCR-171-injected A20 tumors showed significant increases in CD4^+^ T cells and DCs in injected tumors compared to ONCR-159, whereas both oHSVs increased CD8^+^ T and NK cells, as well as gene sets for IFN, cytotoxicity, antigen presentation, and cytokine signaling [29]. Immune depletion studies demonstrated the requirement for CD8^+^ T and NK cells in the A20 model. mONCR-171 efficacy is further enhanced by systemic PD-1 blockade in the MC38 model [29]. What is not clear is whether all the transgenes are contributing to the efficacy observed and whether any are acting antagonistically. ONCR-177 is currently in a phase I clinical trial for advanced solid tumors with surface lesions or liver metastases and combined with anti-PD-1 (NCT04348916).

## 6. Combination Therapies

Chemotherapy, radiation, and molecularly targeted drugs are predominantly employed therapies to treat cancer, often in combination. Due to intratumor heterogeneity and evolution, these treatment modalities are often overcome by resistance. In addition, tumors can evade immunity through multiple mechanisms, such as (i) alteration/loss of antigenic targets, (ii) cell death inhibition, (iii) expression of immunosuppressive agents such as immune checkpoint ligands and cytokines/chemokines, and (iv) downregulation of antigen presentation [173]. Therefore, the combination of oHSVs with other chemotherapeutic and immunotherapeutic agents that can enhance anti-tumor immune responses and efficacy is a potent strategy [2].

### 6.1. Combination with Immune Checkpoint Inhibitors (ICIs)

Immune checkpoint molecules, such as CTLA-4 and PD-1, play critical roles in regulating immune responses and suppressing immune effector cells, thus leading to the development of ICIs, which have proven to be exceptionally effective in some cancers in some patients [174]. Ipilimumab, anti-CTLA-4 antibody, was the first ICI to be approved for clinical use in cancer [174]. PD-1 is expressed on a variety of immune cells, whereas its ligand, PD-L1, is expressed on tumor cells and immune cells. There are multiple potential reasons for patients not responding to ICIs: lack of CD8^+^ T cell tumor infiltration, low tumor cell mutational burden, and low IFNγ signature, or so-called ‘cold’ TME [174]. Infection with oHSV induces an inflammatory response and so-called ‘hot’ TME that should be responsive to ICI, as seen with T-VEC in melanoma patients also treated with anti-PD-1 [175]. The first oHSV + ICI clinical trial was T-VEC combined with ipilimumab in patients with advanced melanoma [176]. This combination resulted in a significantly improved objective response rate (ORR) compared with ipilimumab alone (39% vs. 18%); with responses in non-injected visceral lesions in 52% of patients [177]. In mice, OncoVEX^mGM-CSF^ (mT-VEC) in combination with anti-CTLA-4 significantly extended survival compared to single treatments alone in both A20 and CT-26 bilateral tumor models, with a significant increase in tumor-specific splenocytes [28]. In a phase 1b trial of T-VEC followed by anti-PD-1 (pembrolizumab) in advanced melanoma the CR rate was 33%, with most responding patients lacking CD8^+^ infiltration and an IFNγ signature at baseline, so-called ‘cold’ TME, contrary to what was seen in trials of ICIs alone [175]. Following T-VEC injection there was an increase in CD8 + TILs, and PD-L1 and IFNγ expression [175]. Unfortunately, the recent phase III clinical trial (KEYNOTE-034) of T-VEC in combination with pembrolizumab was stopped due to futility after an interim analysis.

HF10 (Figure 4) was evaluated in a phase II clinical trial in combination with ipilimumab in advanced melanoma with a best overall response (BOR) of 41% [72]. In a phase I/II clinical trial of OH2 (T-VEC-like oHSV-2) alone or in combination with anti-PD-1 (HX008) in patients with advanced solid tumors with injectable lesions, two of 33 patients taking OH2 alone and two of 12 in the combination-treated group had an immune partial response (iPR) that was durable [157]. Increases in PD-L1^+^ cells and CD8^+^ T cells after OH2 alone were observed in most evaluated patients relative to baseline, irrespective of response [157]. Among 40 patients treated with OH2 alone, only two were seropositive for HSV-2 at baseline and 26 seroconverted after treatment [157]. In mouse syngeneic rhabdomyosarcoma models, tumors from a rhabdomyosarcoma cell line expressing high MHC I (M3-9-M) responded to HSV1716 and anti-PD-1, whereas tumors with MHC I low (76–9) did not respond [62]. Interestingly, female mice implanted with male cancer cells responded much better to combination therapy than male mice implanted with the same cells [62].

GBM is an immunosuppressive, ICI non-responsive tumor, where oHSV has demonstrated efficacy in patients [111,124], thus it is reasonable to evaluate whether ICI can enhance oHSV therapy in GBM. In a representative orthotopic mouse GBM stem-like cell model (005), intratumoral G47Δ-mIL12 (see 5.2) combined with systemic anti-PD-1 and -CTLA-4 (triple combination therapy) cured most mice, significantly extending survival compared to mono- or dual-therapies [48]. Triple combination therapy was associated with a significant decrease in CD4^+^ Tregs and an increase in the CD8^+^ T/CD4^+^ Treg ratio, and increased macrophage infiltration and M1-like polarization. Immune cell depletion studies revealed a complex dependency, with CD4^+^ depletion completely abrogating efficacy, and CD8+ and macrophage depletion eliminating all cures [48]. This study demonstrated the need for four components for ‘curative’ therapy of ICI non-responsive tumors; oHSV, local IL-12 expression, anti-PD-1, and anti-CTLA-4, and revealed a complex interconnectedness between immune cells in the TME that makes therapy challenging and difficult to predict active therapeutic targets. It is important to note that the timing of oncolytic virus and ICI administration can impact the therapeutic outcome, although the optimal strategy in the clinic remains to be determined; for example, T-Vec has been given concurrently with, prior to and then concurrently with, or concurrently followed by anti-PD-1 [178].

### 6.2. Combination with Histone Deacetylase (HDAC) Inhibitors

Histone modifications by acetylation play a key role in epigenetic regulation of gene expression and is mainly controlled by the balance between histone deacetylases (HDAC) and histone acetyltransferases (HAT). In human PBMC co-cultures with human melanoma cells, OncoVEX^mGM-CSF^ (mT-VEC) alone induced in vitro features of anti-tumor immunity—IFNα/β, activation of NK cells, and the maturation of DCs and CTLs [179]. The addition of HDAC inhibitor VPA prior (but not concurrently) to virus infection increased virus replication and cytotoxity in human melanoma cells, increased NK ligand expression on melanoma cells and NK cell killing, and CTL priming against melanoma TAAs, all markers of anti-tumor immunity [179]. In contrast, VPA prior to rQNestin34.5 (Figure 4) inhibited NK cell killing of human GBM cells, and decreased early NK cell and macrophage infiltration in human GBM tumors in athymic mice [180]. The effect of NK cells on oHSV anti-tumor activity is a complex balance between inhibition of virus spread early versus NK-cell-mediated cancer cell killing and immunity. The effects of oHSV combinations with HDAC inhibitors in immune competent models remain to be determined.

### 6.3. Combination with MAPK Pathway Inhibitors

Oncogenic, constitutively active mutations in the mitogen-activated protein kinase (MAPK) signaling pathway (RAS → RAF → MEK → ERK) are major drivers in many solid tumors and important targets for drug development [181]. The immunotherapeutic effects of the combination of mT-VEC with MEK inhibition (trametinib) was examined in a syngeneic BRAFV600E melanoma model (D4M3A) [39]. The combination significantly inhibited tumor growth compared to monotherapy, with tumor eradication in ~40% of treated mice, and was associated with a significant influx of effector CD8^+^ T cells, both virus- and tumor-specific. Of the cured mice, 70% were protected from rechallenge with twice the number of D4M3A cells [39]. Depletion of CD8^+^ T cells and Batf3^+^ DCs, but not CD4^+^ T cells or macrophages, abrogated the efficacy of combination treatment. The combination induced an inflammatory gene signature and significant increases in PD-L1^+^ tumor cells and PD-1^+^ immune cells, so that the combination with anti-PD-1 cured six out of seven mice compared with two out of seven for the combination alone [39]. Thyroid tumor cell lines with BRAF^V^600E, generated in transgenic mice and orthotopically-implanted, were sensitive to BRAF inhibitor PLX4720, and somewhat to oHSV mRP1 (see 5.6), which increased effector CD8^+^ TILs [182]. The oHSV + BRAFi combination was more effective in inhibiting tumor growth despite the lack of a combined effect in vitro, which suggests that it is immune-mediated [182]. Depletion of CD8^+^ T cells or NK cells significantly reduced the efficacy of combination treatment. The addition of anti-PD-1 or -CTLA-4 antibodies significantly inhibited tumor growth compared to combination alone or single treatments; over 90% of mice were cured, and these were protected from tumor rechallenge [182].

### 6.4. Combination with TGFβ Inhibitors

TGF-β1 has a diversity of activities in cancer, including driving malignant phenotypes, cancer stem cell maintenance, as well as the suppression of innate and adaptive immune cells [183]. In two syngeneic murine rhabdomyosarcoma models, monotherapy with HSV1716 or TGF-βR1 inhibitor (A8301) alone resulted in no or modest improved efficacy over controls. However, combination therapy, despite having no effect in vitro, significantly prolonged survival, including ~20% complete durable responses [63]. This improved therapeutic efficacy of combination therapy was T-cell-dependent [63]. Although combination treatment greatly increased CD8^+^ TILs and the CD8^+^/Treg ratio, it was not different from HSV1716 alone [63]. A contrary study in a syngeneic mouse GBM model (4C8) showed that TGFβ1 administration prior to rQNestin34.5 prolonged survival, whereas anti-TGFβ antibody (ID11) abrogated the effect of rQNestin34.5 alone, which was also seen in a human GBM stem-like cell xenograft model [49]. The effect of TGFβ was due to inhibition of NK cells and macrophage infiltration and activation [49]. Another study in a human GBM stem-like cell xenograft model (MGG31) contradictorily found that TGF-βR1 inhibitor (galunisertib) synergized with oHSV MG18L in extending survival, with 60% cures [121].

## 7. Future Priorities for Therapeutic Development

When OVs were first used in humans there was real concern about safety and other pathogenic effects, but the experience has been that G207, G47Δ, T-VEC, and other oHSVs have generally been safer than many other drug therapies and therapeutic antibodies [13,108,111]. The priorities are now to improve: (i) intratumoral delivery techniques of oHSVs and target distant tumors; (ii) replication and spread of the virus within the tumor; (iii) systemic immune responses against tumor antigens exposed by virus infection; and (iv) proinflammatory TME. Optimizing the intratumoral delivery of more potent oHSVs will result in better TME reprogramming and remodeling, and anti-tumor immunity.

Direct administration of oHSV via intratumoral delivery is the most common and possibly the best route because of high anti-HSV antibody prevalence (seropositivity) in humans and studies demonstrating the abscopal effects of oHSV-injected tumors on non-injected distal lesions, which is the essence of an effective ISCV. Currently, many aggressive malignant cancers are refractory to standard-of-care treatments, and we lack effective alternatives. In those cases, oHSV-mediated ISCV for those tumors accessible to intratumoral delivery is a warranted approach. It is important to focus on tumor biology and what attributes may be targetable by oHSV. For example, brain tumors are not easily accessible to many drugs or systemically delivered oHSVs because of the blood–brain barrier, so intratumoral injection may be preferable. Studies have shown that multiple inoculations can act as a “booster” shot and provide increased efficacy versus a single inoculation of oHSV, as with T-VEC [153]. Indeed, it was shown that as many as six intracranial inoculations of G47Δ were safe and well-tolerated in patients [124]. For more disseminated tumor metastases, abscopal effects will be even more important and multiple tumors may need to be targeted. This highlights some of the ongoing challenges in the field—how to improve ISCVs and develop strategies for the systematic administration of oHSVs that will be safe and effective.

Optimizing oHSV replication, immunogenic cell death, and spread within the tumor is critical for improving oHSV efficacy. Armed oHSVs also act as gene therapy vectors to expand the range of therapeutic targets and impact non-infected cells and the TME [12]. One strategy to improve their spread is to express or inject extracellular-matrix-degrading enzymes in the tumor [184]. Recently, an oncolytic adenovirus expressing hyaluronidase was shown to enhance efficacy in an immunocompetent mouse GBM model by modifying the TME, inducing T cell infiltration and M1-like polarization of infiltrating macrophages [185]. We and other researchers are continually searching for the most potent oHSV that is safe and efficacious at killing tumor cells and inducing antitumor immunity, and then combining them with immunomodulatory agents or transgenes that will amplify immunity. A turn-off or modulation mechanism could avoid major adverse events in patients. The unrestricted replication of oHSV can be prevented by current antiviral drugs such as acyclovir or its analogs [186], but we currently do not have turn-off strategies for major immune-related adverse events.

Most immune-based therapeutic approaches, such as ICI, require a pre-existing immune response to the patient’s cancer to have an effect. Typically, a minority of patients respond to systemic anti-PD-1 or PD-L1 even in ICI-responsive cancers, and it is important to recognize that these response rates and durability need to be improved. Modalities to induce effective anti-tumor immune responses are limited. Oncolytic immunovirotherapy is one such strategy, which can be combined with other forms of immunotherapy to potentiate the immune response, synergizing and providing efficacy that is better than either agent alone. oHSVs can potentially turn a patient’s immune status from a so-called ‘cold’ to ‘hot’ TME that is more likely to respond to ICI immunotherapy. The safety profiles for oHSVs have been quite good and typically better than many other therapeutics. There are two core challenges to immunotherapy: (i) improving outcomes and overcoming resistance in immunotherapy-responsive cancers, and (ii) making ‘cold’ non-responsive tumors ‘hot’. Choosing the right preclinical models that represent the patient’s disease, which are often difficult to treat, is important in order to develop clinically translatable therapies. The formulation, dosing, and scheduling of oHSV and immunotherapeutic agent administration need to be optimized.

Another challenge for the field is to increase the biological information obtained in clinical trials. Thus far, some impressive durable responses have been observed with oHSVs, even in highly malignant tumors, but overall response rates remain low. How do we identify why some patients respond and others do not? Patient stratification will require identifying biomarkers to better understand which patients might respond to oHSV therapy. It will be important to take sampling into account when designing accrual and biopsy-intensive clinical trials so that there are enough patient samples to investigate potential baseline gene signatures or biomarkers of patients more likely to respond to oHSV.

## 8. Conclusions

OHSV provides a novel therapeutic modality for cancer. It is usually genetically-engineered for safety, selective replication, and cytotoxicity in cancer cells. Selectivity is based on cancer cell physiology and the identification of viral genes that are dispensable in cancer, but not normal cells or receptor-retargeting viral glycoproteins to interact with cancer-specific cell surface molecules. Initially, oHSVs were developed to directly kill cancer cells and spread in the tumor. However, further studies demonstrated the robust ability of oHSV-infected tumor cells to induce anti-tumor immune responses and ISCV, so much of the focus on oHSV development has shifted to enhancing immunovirotherapy, mostly through arming oHSVs with immunomodulatory transgenes or combining them with immunomodulatory therapeutics. The evolving landscape of oHSV-mediated immunovirotherapy is intense now in terms of the number of different oHSVs in clinical trials and companies investing in the technology. This has been spurred by the FDA and EMA approval of T-VEC for advanced melanoma, as the first oncolytic virus approved in the USA and Europe [5]. It is important for the field that we continue to see safety and improved efficacy in clinical trials with various oHSVs and various cancer types.

## Figures and Tables

**Table 1:** Immunocompetent mouse cancer cell tumor models treated with oHSV.

Cancer Cell Line	Tumor Type	Mouse Strain	oHSV	References
CT26	Colon carcinoma	BALB/c	G207	[6,25,26]
			NV1020	[26]
			oHSV2	[19]
			OV-IL15C	[27]
			OncoVex^mGMCSF^	[28]
			mONCR-171	[29]
MC26	Colon cancer	BALB/c	HF10	[30]
			hrR3	[31]
MC38	Colon cancer	C57Bl/6	mONCR-171	[29]
N18	Neuroblastoma	A/J	G207	[24,32]
Neuro-2a	Neuroblastoma	A/J	G207	[33]
			G47Δ	[33]
			C134	[34]
			M002	[35]
S91 Cloudman M3	Melanoma	DBA/2	G207	[6]
			HSV1716	[36]
			HF10	[37]
Harding–Passey	Melanoma	C57Bl/6	HSV1716	[38]
D4M3A	Melanoma	C57Bl/6	mT-VEC	[39]
B16R	Melanoma	C57Bl/6	oHSV2-aPD1	[40]
M6c	Breast cancer	C3(1)/T-Ag	G47Δ	[41]
4T1	Breast cancer	BALB/c	oHSV2	[42]
			G47Δ-IL12	[43]
			FusOn-H2	[44]
			oHSV2	[42]
			HSV1716	[45]
			ΔN146	[46]
MMTV-PyMT-Ts1	Breast cancer	FVB	HSV1716	[45]
005	Glioblastoma	C57Bl/6	G47Δ	[47]
			G47Δ-IL12	[48]
CT2A	Glioblastoma	C57Bl/6	G47Δ-IL12	[48]
DBT	Glioblastoma	BALB/c	C134	[34]
4C8	Glioblastoma	B6D2F1	rQNestin34.5	[49]
			M002	[50]
GL261N4	Glioblastoma	C57Bl/6	NG34scFvPD-1	[51]
SCC-VII	Squamous cell carcinoma	C3H/HeN	HF10	[52]
			NV1020, NV1042	[53]
TC-1	HPV+ cervical cancer	C57Bl/6	G47Δ (T-01)	[54]
Hepa1-6	Hepatoma	C57Bl/6	G47Δ (T-01)	[55]
			OVH-aMPD1	[56]
TRAMP-C2	Prostate	C57Bl/6	NV1023, NV1042	[57]
MBT-2	Bladder	C3H/He	FusOn-H2	[58]
A20	Lymphoma	BALB/c	OncoVex^mGMCSF^	[59]
			VG161	[60]
			mRP2	[61]
			mONCR-171	[29]
76-9	Rhabdomyosarcoma	C57Bl/6	HSV1716	[62,63]
M3-9-M	Rhabdomyosarcoma	C57Bl/6	HSV1716	[62,63]
